# Characterization and anti-oxidant properties of powder from differently dried beetroot (*Beta vulgaris* L.) slices

**DOI:** 10.3389/fnut.2026.1939515

**Published:** 2026-09-18

**Authors:** Kanupriya Choudhary, Subir Kumar Chakraborty, Debabandya Mohapatra, Rajendra Hamad

**Affiliations:** 1 ICAR - Central Institute of Agricultural Engineering, Bhopal, India; 2 Department of Processing and Food Engineering, College of Technology and Agricultural Engineering, Agriculture University, Jodhpur, India; 3 ICAR - National Institute of Secondary Agriculture, Ranchi, India

**Keywords:** beetroot slice, betalain retention, freeze drying, microwave vacuum drying, powder characteristics

## Abstract

**Objective:**

This research work was aimed at investigating the changes in phytochemical constituents during the dehydration of beetroot slices using microwave vacuum drying and freeze drying. The results are reported in terms of the overall quality attributes of the resulting powder from the beetroot slices.

**Methods:**

Powder characteristics and antioxidant properties of beetroot (*Beta vulgaris*) slices of varying (1 and 2 mm) thickness dried under microwave (power, 160 and 320 W) vacuum (710, 660 and 610 mm Hg, absolute pressure) drying (MVD) and freeze drying (FD) (0.062 mbar chamber pressure and -42 °C condenser temperature) were investigated in this study. Quality of the beetroot powder was evaluated based on moisture content, water activity, colour attributes, powder properties, functional characteristics, antioxidant activity and betalain content.

**Results:**

Significant (*p* ≤ 0.05) difference was observed across all the parameters under the different drying conditions. MVD significantly reduced drying time while producing powder with high antioxidant activity, porosity an improved solubility. However, FD samples for lower thickness (1 mm) exhibited superior retention of colour, and betalain pigments due to low temperature processing but antioxidant activity was found 24.51% RSA. Among MVD conditions, higher microwave power (320 W) combined with moderate vacuum ≤660 mm Hg resulted in an improved retention of bioactive compounds, colour quality and %RSA (60-72%) for the powder.

**Conclusions:**

The findings of the study highlight that MVD can be effectively utilized as an alternative for FD, as a low cost drying for the production of pigment enriched beetroot powder for versatile commercial food colouring applications.

## 1 Introduction

Beetroot (*Beta vulgaris* L.) is a root vegetable belonging to the Chenopodiaceae family, widely recognized for its rich composition of bioactive compounds, particularly betalain pigments and phenolic compounds (1). These constituents contribute significantly to its antioxidant, anti-inflammatory and therapeutic properties. In recent years, beetroot and its derived products, especially juice have gained attention as functional foods due to their potential role in managing chronic conditions such as type 2 diabetes, hypertension, hypercholesterolemia and certain pulmonary disorders (2). Additionally, beetroot based snack products have emerged in the market as valuable sources of dietary fibre, essential fatty acids and proteins. The presence of betalains (betacyanins and betaxanthins) and total phenolics enhances the antioxidant capacity of beetroot, which is associated with antiatherosclerotic, anticancer, and antitumor activities (3). Moreover, betalain pigments are increasingly utilized as natural colorants in food systems such as confectionery, dry mixes, jams and jellies due to their vibrant colour and safety compared to synthetic dyes (4). However, these pigments are highly sensitive to environmental and processing conditions, particularly temperature, oxygen and light. Conventional drying methods involving high temperatures and prolonged durations can cause substantial degradation of pigments and reduce antioxidant potential. In response to these limitations, advanced drying technologies have been developed to enhance the retention of thermosensitive compounds and ensure product quality (5, 6).

Drying is one of the oldest and most widely used preservation techniques for fruits and vegetables. Conventional drying methods such as solar drying, tray drying, drum drying and freeze drying are commonly employed for producing powder and chips, same has been reported with beetroot also (7, 8). However, drying conditions play an important role in determining the final quality of dried product, mostly the retention of bioactive compounds responsible for health promoting effects (9). Freeze drying (FD), also known as lyophilisation, is a drying method in which first food is frozen then dried by direct sublimation of ice by marinating a temperature and pressure conditions below triple point of water in drying chamber. Freeze drying is considered as best method for producing high quality dried food products. But at the same time, it involves a high production cost, high energy consumption and low throughputs (10). Microwave vacuum drying (MVD) has emerged as a promising alternative drying technique due to its ability to combine rapid volumetric heating with low temperature processing under vacuum conditions. In this method, microwave energy interacts with water molecules (dipole rotation), generating internal heat and creating pressure gradients that facilitate rapid moisture removal (11). The application of vacuum lowers the boiling point of water, enabling drying at reduced temperatures, thereby minimizing thermal degradation of sensitive compounds. Consequently, MVD offers significant advantages over conventional drying methods, improved retention of bioactive compounds and better preservation of structural and sensory attributes. Among the various process parameters, microwave power level plays a critical role in influencing input energy use efficiency and overall product quality (12). MVD has been effectively applied to various food products due to its ability to improve drying efficiency while preserving product quality. In orange slices, MVD reduced drying time and improved retention of carotenoids, vitamin C and colour quality by minimizing thermal and oxidative degradation under vacuum conditions (13). Similarly, in berries, MVD preserved anthocyanins, phenolics, and antioxidant activity due to rapid volumetric heating and oxygen deficient vacuum conditions that enhanced pigment stability and colour retention (14). In okra, MVD improved drying efficiency while preserving chlorophyll, phenolic compounds and antioxidant properties, along with reduced shrinkage and structural damage (15). Comparable findings were reported for jujube, where MVD enhanced retention of phenolics, flavonoids and vitamin C due to shorter drying time and lower oxygen exposure (16). Since betalains in beetroot are highly sensitive to heat, oxygen and prolonged drying, the low temperature and oxygen deficient conditions of MVD can be a suitable alternative for beetroot processing leading to beetroot powder making in particular. Additionally, rapid microwave heating minimizes cellular damage and pigment oxidation, thereby improving betanin and total betalain retention. Therefore, MVD can be considered a promising drying technology for producing high-quality beetroot powder with improved pigment stability, nutritional value, and colour retention.

The reported research work is aimed at investigating the changes in phytochemical constituents during the dehydration of beetroot slices using microwave vacuum drying, efficiency of the drying process in the retention of pigments in beetroot powder. Furthermore, the microwave power and vacuum levels employed in the present study have not been previously reported for the drying of beetroot. In addition, the study provides a comparative evaluation of microwave vacuum drying and freeze drying based on the overall quality attributes of the resulting beetroot powder.

## 2 Materials and methods

### 2.1 Materials and sample preparation

All chemicals and reagents used in this study were of analytical grade and procured from HiMedia. Fresh beetroot (*Beta vulgaris* L., var. NSC Red) was cultivated and harvested from the experimental field 23.315822° N, 77.400955° E of ICAR-Central Institute of Agricultural Engineering (CIAE), Bhopal, India. Immediately after harvesting, the beetroot samples were thoroughly washed under running tap water to remove adhering soil particles and surface impurities. The cleaned samples were manually peeled and subsequently sliced into uniform thickness using a pedal operated potato slicer developed at ICAR- CIAE to ensure consistency in sample geometry. The prepared slices were immediately subjected to drying experiments to minimize enzymatic degradation and quality deterioration prior to processing. All experiments were conducted in triplicate to ensure reproducibility and the prepared samples were stored under controlled conditions prior to analysis.

### 2.2 Drying

Drying was conducted to decrease the moisture content of fresh sliced beetroot thereby enabling powder making for further experimental use. MVD was employed for rapid moisture removal under reduced pressure to minimize thermal degradation and retain heats sensitive compounds, while freeze drying served as a reference method due to its characteristic preservation capabilities of maintaining structural, colour, and nutritional attributes of products.

#### 2.2.1 Microwave vacuum drying

The microwave vacuum drying laboratory setup was developed at ICAR-CIAE, Bhopal, India. In MVD system, vacuum was generated using an external vacuum pump connected to the drying chamber through a vacuum desiccator. The desired vacuum level was regulated using the vacuum valve of the desiccator, while a vacuum gauge mounted on the pump monitored the chamber pressure throughout drying. This arrangement maintained stable vacuum conditions during experiments. Drying experiments were conducted by varying three key process parameters: slice thickness (1 and 2 mm), microwave power (160 and 320 W), and vacuum pressure (710, 660, and 610 mm Hg, expressed as absolute pressure). These experimental levels were selected based on preliminary trials conducted to identify suitable operating ranges for the microwave vacuum drying of beetroot slices. During the drying process, the generated water vapour from beetroot slices was continuously removed using a condenser unit to maintain the desired vacuum conditions and enhance mass transfer. Based on preliminary trials and visual quality assessment under different combinations of microwave power and vacuum levels, appropriate exposure times were optimized for each treatment to achieve equilibrium moisture content. Upon completion of drying, the samples were cooled to ambient conditions and stored in airtight amber glass containers to prevent moisture reabsorption and photo degradation prior to further analysis.

#### 2.2.2 Freeze drying

Freeze drying of beetroot slices (1 and 2 mm) was carried out at deep freezing temperature of −20 ± 2 °C for 24 h to ensure complete solidification of water within the tissue matrix. The frozen samples were then placed in the sample holder of a laboratory scale freeze dryer (Mini-Lyodel-LYO1550, Delvac Pumps Pvt. Ltd., Chennai, India). Freeze drying was continued until a constant sample weight was achieved. For this, sample and holder were weighed before drying and periodically reweighed by temporarily stopping the freeze dryer. Drying was considered complete when successive measurements, initially at 1 h and subsequently at 30 min intervals, showed negligible weight variation. The dried slices were then removed from the holder, powdered and subjected to subsequent analyses. The drying process was carried out under controlled and constant vacuum conditions (0.062 m bar), facilitating sublimation of ice directly into vapour.

### 2.3 Moisture content and water activity

Moisture content of the samples was determined according to the standard method (17). The moisture content was calculated using Equation 1 based on the weight difference before and after drying. The water activity (a_w_) of the beetroot powder obtained from different drying treatments was measured using a water activity meter (WA-160A, Amittari, Guangdong, China). All measurements were performed under controlled conditions and readings were recorded after achieving equilibrium within the instrument chamber.

$$\text{Moisture Content}\;(\%\mathrm{wb})=\frac{W_{1}-W_{2}}{W_{1}}\times 100$$ (1)

Where, W_1_ is the initial weight of the sample, g and W_2_ is the final weight of the sample, g after drying.

### 2.4 Powder characteristics

The beetroot powder was characterized to assess the effect of drying methods on its properties related to quality (molecular structure and colour) and functional (Carr index, Hausner ratio and reconstitution properties) attributes interpreted as flowability indices were also determined to assess handling characteristics and suitability of the beetroot powder for food applications.

#### 2.4.1 Functional characterization of powder particles

The dried slices were ground into powder using an analytical mill (4301-02, Cole-Parmer Instruments Co., India). The resulting powder were sieved to obtain a uniform particle size fraction in the range of 200–350 μm using standard BSS sieves (No. 50 and 70). The sieved powder samples obtained from different drying treatments were subjected to Fourier Transform Infrared (FTIR) spectroscopy to identify characteristic functional groups and evaluate molecular alteration in bioactive constituents through analysis of absorption band in the infrared spectra. FTIR measurements were carried out using a spectrometer (NICOLET 6700 Thermo Fisher Scientific, Germany) equipped with compatible software (OMNIC, version 6.1). A small amount of beetroot powder obtained from MVD and FD was separately mixed with KBr and carefully put into a pellet-forming mould, and squeezed under hydraulic pressure, with an average of 32 scans, the infrared absorption of thin film between 4,000 to 400 cm^−1^ was measured. All spectra were taken at room temperature and under natural light (18).

#### 2.4.2 Colour measurement

The colour characteristics of beetroot powder were measured using a colorimeter (CS-10, Amtast, Guangdong, China). Initially, the instrument was calibrated using the standard calibration plates provided by the manufacturer to ensure accuracy and reproducibility. Colour values were recorded in the CIE L*a*b colour space, where L represents lightness, a denotes the red/green coordinate and b indicates the yellow/blue coordinate. The total colour difference (ΔE) and Browning index was calculated (19), using Equations 2–4.

$$\text{Colour change}\Delta E=\sqrt{\Delta L^{2}+\Delta a^{2}+\Delta b^{2}}$$ (2)

Where, ∆L = (L_0_-L), ∆a = (a_0_-a) and ∆b = (b_0_-b) and L_0,_ a_0_ and b_0_ are values of fresh beetroot sample, while L, a and b correspond to the dried samples.

$$\text{Browning Index}\;(\mathrm{BI})=\frac{\left(100(X-0.031)\right)}{0.17}$$ (3)

Where,

$$X=\frac{\left(a+1.75L\right)}{\left(5.645L+a-3.012b\right)}$$ (4)

#### 2.4.3 Carr index, Hausner ratio and bulk porosity

About 10 g of beetroot powder was taken to determine the carr index, bulk porosity and Hausner ratio based on tapped density (TD, g/mL), Particle density (PD, g/mL)and bulk density (BD, g/mL) value (20), using Equations 5–7.

$$\mathrm{CI}=(\frac{\mathrm{TD}-\mathrm{BD}}{\mathrm{TD}})\times 100$$ (5)

$$\mathrm{HR}=\frac{\mathrm{TD}}{\mathrm{BD}}$$ (6)

$$\mathrm{BP}(\text{Bulk Porosity})=(1-\frac{\mathrm{BD}\;}{\mathrm{PD}})\times 100$$ (7)

#### 2.4.4 Reconstitution properties

The evaluation of reconstitution properties provides insight into the functional performance of dried powder during rehydration. WAI represents the capacity of powder particles to absorb and retain water, whereas WSI measures the degree of solubilization of powder constituents in water. About 1 g of beetroot powder was taken to determine WAI and WSI (21). WAI and WSI values are calculated by using Equations 8, 9.

$$\mathrm{WSI}\;(\%)=\frac{\;\text{Weight of dried supernantant}\;(g)}{\text{Weight of beetroot powder}\;(g)}\times 100$$ (8)

$$\mathrm{WAI}(\frac{g}{g\;\text{of}\;\mathrm{dry}\;\text{matter}})=\frac{\text{Weight of}\;\mathrm{wet}\;\text{sediment}\;(g)}{\text{Weight of beetroot powder}\;\;(g)}$$ (9)

### 2.5 Biochemical parameters

The beetroot powder was analysed to evaluate the effect of drying methods on its biochemical and nutritional quality attributes. Proximate analysis was carried out to determine the nutritional composition, while total flavonoid content, antioxidant activity, and betalain content were assessed to evaluate the retention of bioactive compounds and antioxidant potential during drying.

#### 2.5.1 Proximate analysis

The proximate composition of beetroot powder, including protein, carbohydrate, crude fibre, fat, and ash content, was determined according to standard methods (17). The total energy value (EV) of the samples was calculated using the Atwater general factor (Equation 10). Additionally, all analyses were performed in triplicate, and the results were reported on a dry weight basis to ensure accuracy and reproducibility.

$$\begin{array}{l} \mathrm{EV}\;(\text{kcal})=\text{Protein}(\%)\times 4+\text{Carbohydrate}\;(\%)\\ \times 4+\mathrm{Fat}\;(\%)\times 9 \end{array}$$ (10)

#### 2.5.2 Total flavonoid

About 1 g of beetroot powder was extracted with 20 mL of 80% (v/v) aqueous methanol. The mixture was subjected to sonication for 15 min (twice) to enhance extraction efficiency, followed by incubation at 4 ± 1 °C for 24 h in dark conditions to prevent degradation of light sensitive compounds. Subsequently, the extract was centrifuged at 3500 rpm for 10 min at 4 °C, and the supernatant was collected and filtered through Whatman No. 1 filter paper to obtain a clear extract (22). The TFC was quantified using a catechin standard calibration curve and expressed as micrograms of catechin equivalents (μg CE/g dry matter).

#### 2.5.3 Antioxidant activity

Free radical scavenging activity (RSA) of the sample was determined using the DPPH assay (23). Absorbance was measured at 517 nm, RSA (%) was calculated using the following Equation 11.

$$\begin{array}{l} \text{Radical scavenging activity}\;()=\\ \;\;\;\;\;\;\;\;\;\;\frac{{\text{Absorbance}}_{\text{control}}-{\text{Absorbance}}_{\text{sample}}}{{\text{Absorbance}}_{\text{control}}}\times 100 \end{array}$$ (11)

#### 2.5.4 Extraction and estimation of betalains

About 0.1 g of dried beetroot powder was mixed with 20 mL of distilled water. This mixture was then centrifuged (C 24, Remi Group Laboratory Instruments, India) for 10 min at 3300 RPM. The supernatant was collected by filtering the extract using qualitative filter paper (100,125 R, Axiva Sichem Biotech, India) and the insoluble were re-extracted two more times. Finally, the supernatants were combined to make up a volume of 50 mL with distilled water (24). Betanin and betaxanthin content of the extracts were determined spectrophotometrically at 538 nm (A_538_) and 476 nm (A_476_) respectively (Equation 12). The betalain content was calculated as

BC(mg/L)=(A×DF×MW×1000)/(e×L) (12)

Where, A: Absorption, DF: Dilution Factor and L is the path length (1 cm) of the cuvette.

For quantification of betanin (betacyanin) and betaxanthins (vulgaxanthin-I), the molecular weights (MW) of 550 and 308 g/mol respectively, and molar extinction coefficients (e) of 60,000 and 48,000 L/mol-cm in water used.

### 2.6 Statistical analysis

All experiments were conducted using a full-factorial completely randomized design with three replications. The experimental data were analysed using student edition of Design Expert® software (Version 13.0.5.0, Stat-Ease Inc., Minneapolis, USA). Analysis of variance (ANOVA) was performed to determine the significance of the independent variables. Also, Tukey’s test was applied to compare the means of significant factors. Statistical significance was considered at *p* ≤ 0.05.

## 3 Results and discussion

This section features a focused approach in evaluating the effect of different drying methods on the quality characteristics of beetroot powder. Moisture content and water activity were analysed to assess drying efficiency and storage stability, while powder characteristics were determined to evaluate the handling and reconstitution properties. Also reported in the biochemical analysis including proximate composition, total flavonoid content, antioxidant activity, and betalain content as parameters to examine the retention of nutritional and bioactive compounds in the beetroot powder after drying.

### 3.1 Moisture content

The initial moisture content of fresh beetroot was determined as 87.10 ± 0.5% (%wb). The moisture content of beetroot slices during MVD was significantly affected by microwave power, vacuum level and slice thickness. The moisture content of 5% or less is consider safe for fruit powders, as it is essential for improving stability and extending the shelf life of food powders (25). The minimum moisture content (1.55%) was observed at 320 W, 160 mm Hg and 1 mm slice thickness with minimum drying time (12.67 min). However, increasing the slice thickness to 2 mm extended the drying time to 16.00–16.67 min but able to maintain a moisture content below 5%. At lower power also (160 W) moisture content of dried powder was also maintained below 5% but drying time increased (25.33–38.00 min) further which indicates a reduced drying efficiency. Higher microwave power and reduced slice thickness enhanced drying rate due to rapid volumetric heating and shorter moisture diffusion paths, while vacuum conditions facilitated moisture removal. However, excessive power and vacuum may increase product temperature and induce structural collapse, leading to degradation of heat-sensitive compounds (26). Freeze drying required significantly longer processing time to achieve similar moisture levels (27). In this light, it can be inferred that MVD provides a rapid and efficient drying approach, but optimal parameter selection is essential to balance drying rate and product quality (28). Moisture content varied significantly among the drying treatments (*p* < 0.05), ranging from 1.55 ± 0.03 to 3.65 ± 0.14%. The superscript letters indicate significant differences among treatments, with treatments bearing the same letter showing no significant difference.

### 3.2 Water activity

Water activity (a_w_) is an important parameter governing the microbial stability, physicochemical properties, and overall quality of food products. In the present study, a_w_ values decreased progressively with drying time under MVD. The increase in microwave power level enhanced the drying rate due to elevated product temperature and rapid moisture removal. MVD enabled in reduction in a_w_ to below 0.5, ultimately achieving final a_w_ in the range of 0.35–0.44 across different combinations of power, vacuum levels and slice thicknesses of beetroot (Table 1). These values indicate improved shelf stability and reduced susceptibility to microbial spoilage.

**Table 1:** Drying conditions and slice thickness induced effect on beetroot powder.

Drying parameter		Beetroot slice		Beetroot powder	
Power level (W)	Vacuum (mm Hg)	Thickness (mm)	Drying time (min)	Moisture content (%)	Water activity (a_w_)
Microwave vacuum drying					
160	710	1	37.00 ± 2.00ᵃ	2.49 ± 0.02ᵃ	0.42 ± 0.01ᵃ
320	710	1	14.00 ± 0.00ᶠ	1.76 ± 0.03ᶠ	0.39 ± 0.01ᶜ
160	660	1	31.33 ± 1.53ᵇ	2.02 ± 0.04ᶜ	0.40 ± 0.01ᵇ
320	660	1	13.17 ± 0.29ᶠ	1.58 ± 0.02ʰ	0.37 ± 0.01ᵉ
160	610	1	25.33 ± 1.53ᶜ	1.65 ± 0.05ᵍ	0.38 ± 0.01ᵈ
320	610	1	12.67 ± 0.58ᶠ	1.55 ± 0.03ʰ	0.35 ± 0.01ᶠ
160	710	2	38.00 ± 1.00ᵃ	3.65 ± 0.14ᵃ	0.44 ± 0.01ᵃ
320	710	2	16.67 ± 1.15ᵉ	2.29 ± 0.07ᵈ	0.41 ± 0.01ᵇ
160	660	2	32.00 ± 1.00ᵇ	2.73 ± 0.39ᵇ	0.42 ± 0.01ᵃ
320	660	2	16.33 ± 0.58ᵉ	2.11 ± 0.04ᵉ	0.39 ± 0.01ᶜ
160	610	2	26.00 ± 1.00ᶜ	2.22 ± 0.02ᵈ	0.40 ± 0.01ᵇ
320	610	2	16.00 ± 1.00ᵉ	1.94 ± 0.06ᶠ	0.37 ± 0.01ᵉ
Freeze drying (0.062 m bar, −40 °C)		1	1950.0 ± 30.0ᵇ	2.37 ± 0.31ᵈ	0.32 ± 0.01ᵍ
		2	3154.0 ± 30.8ᵃ	3.13 ± 0.15ᵇ	0.36 ± 0.02ᶠ

Different superscript small letters in each column denote significant difference at *p* ≤ 0.05.

Water activity is inherently temperature-dependent, and prolonged exposure to elevated temperatures can induce structural modifications in the food matrix (29). Additionally, the interaction of water with hydrophilic constituents results in hydrogen bonding, leading to hydration and conformational changes in macromolecules, which in turn influences water mobility and binding behaviour (30). Such changes may also impact pigment stability and overall product quality. The observed rapid reduction in a_w_ under MVD conditions is consistent with previous findings, where higher drying rates facilitated efficient moisture removal and improved product stability (19). Water activity also differed significantly among the treatments (*p* < 0.05), ranging from 0.32 ± 0.01 to 0.44 ± 0.01. Treatments with different superscript letters were significantly different, while those sharing the same letter were statistically similar.

### 3.3 Powder characteristics

#### 3.3.1 FTIR analysis

The FTIR spectra of microwave vacuum dried (MVD) beetroot slices of 1 and 2 mm thickness depicted noticeable variations in the retention of functional groups associated with betalains, phenolics, flavonoids, ascorbic acid, carbohydrates, and other antioxidant compounds. The major absorption bands observed at 1000–1500, 1,500–1700, 2,800–3,000, and 3,200–3,600 cm^−1^ corresponded to C–O/C–O–C stretching, aromatic C=C and amide I vibrations, aliphatic C–H stretching, and O–H stretching vibrations, respectively (Figure 1).

**Figure 1:**
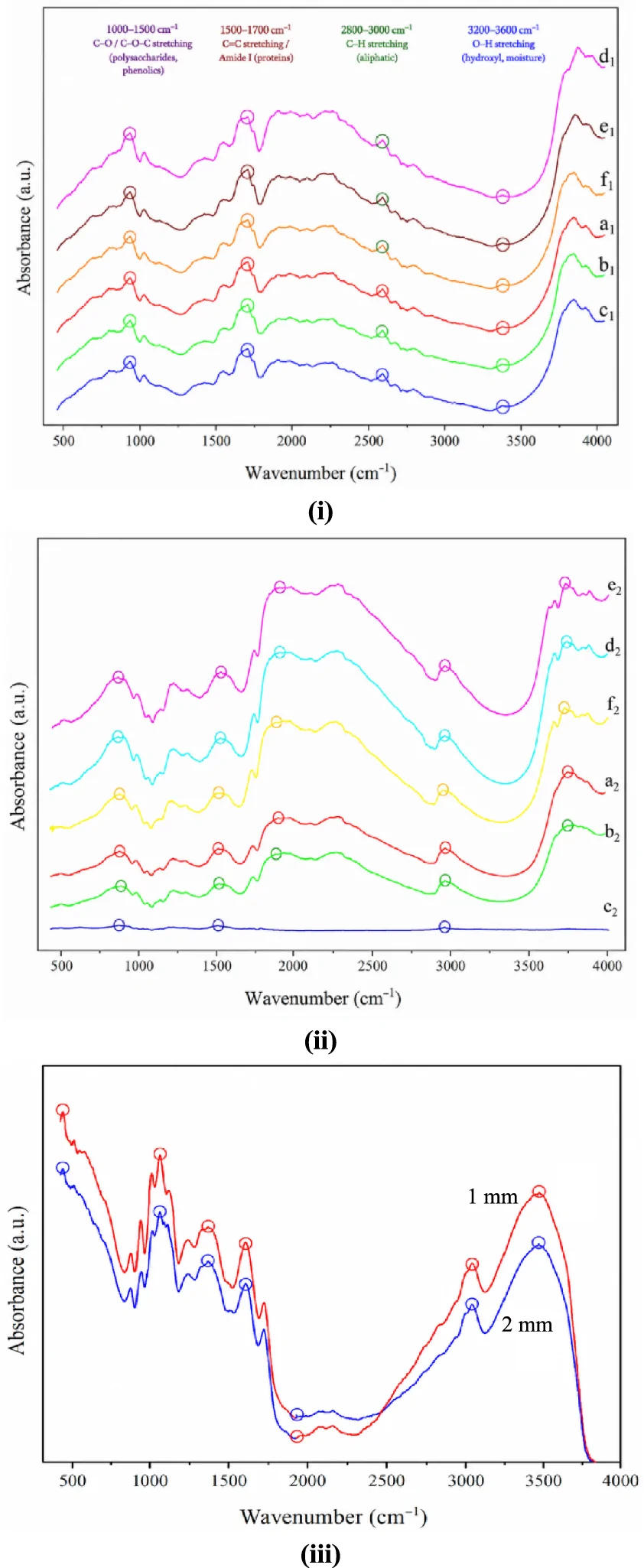
FTIR spectra of beetroot powder under **(i)** microwave vacuum drying, slice thickness 1 mm **(ii)** microwave vacuum drying, slice thickness 2 mm **(iii)** freeze drying for 1 and 2 mm slice thickness (a = 160 W, 710 mm Hg, b = 160 W, 660 mm Hg, c = 160 W, 610 mm Hg, d = 320 W, 710 mm Hg, e = 320 W, 660 mm Hg, f = 320 W, 610 mm Hg).

For the 1 mm slices (Figure 1i), sample d₁ (320 W, 710 mm Hg) exhibited the highest absorbance intensity in all major spectral regions, indicating maximum retention of bioactive compounds and functional groups. The strong O–H stretching band suggested better preservation of hydroxyl-containing compounds such as phenolics, flavonoids, betalains, and ascorbic acid, while the pronounced peaks in the 1,000–1,500 cm^−1^ region indicated greater retention of polysaccharides and betalain-associated structures. The improved retention at higher microwave power may be attributed to rapid volumetric heating and shorter drying duration, which minimized thermal degradation and oxidation of thermosensitive compounds (31). Samples e₁ and f₁ also showed comparatively higher absorbance than the 160 W treatments, whereas c₁ (160 W, 610 mm Hg) exhibited the lowest peak intensity, indicating greater degradation of antioxidant compounds due to prolonged exposure to heat and oxygen during slower drying (32). The 2 mm slices showed (Figure 1ii) similar functional groups but comparatively lower absorbance intensity than the 1 mm slices, indicating reduced retention of thermosensitive compounds due to increased slice thickness. Among the 2 mm treatments, e₂ (320 W, 660 mm Hg) showed the highest absorbance intensity, followed by d₂ (320 W, 710 mm Hg) and f₂ (320 W, 660 mm Hg), indicating better preservation of phenolics, flavonoids, betalains and ascorbic acid. The lower retention observed in thicker slices may be due to higher internal moisture diffusion resistance and prolonged heating of inner tissues, which promoted degradation of sensitive compounds (33). In contrast, weaker absorption bands observed in a₂ (160 W, 710 mm Hg), b₂ (160 W, 660 mm Hg), and particularly c₂ (160 W, 610 mm Hg) suggested comparatively greater degradation during drying. Overall, the FTIR spectra demonstrated that treatments processed at 320 W showed better retention of functional groups and antioxidant compounds than the 160 W treatments. Rapid moisture removal under optimized microwave vacuum conditions have been reported to have reduced oxidation and structural collapse of bioactive compounds in essential oil extracted from sweet orange (31). Based on spectral intensity, d₁ exhibited maximum retention among the 1 mm slices, while e₂ showed the highest retention among the 2 mm slices. Furthermore, the 1 mm slices exhibited superior preservation of bioactive compounds compared to the 2 mm slices due to faster moisture removal and lower thermal stress during drying (32).

Among the FD samples (Figure 1iii), a₁ (1 mm) exhibited higher absorbance intensity than a₂ (2 mm) across all major spectral regions, indicating better retention of bioactive compounds in thinner slices. The stronger O–H stretching band suggested improved preservation of phenolics, flavonoids, betalains, and ascorbic acid, while the prominent peaks in the 1,000–1,500 cm^−1^ region indicated greater retention of polysaccharides and betalain-associated structures. The superior retention in FD samples may be attributed to low-temperature moisture removal through sublimation, which minimized thermal degradation of sensitive compounds (33).

Comparative analysis of MVD and FD demonstrated that both methods preserved the major functional groups of beetroot. However, optimized MVD treatments, particularly d₁ (320 W, 710 mm Hg) and e₂ (320 W, 660 mm Hg), exhibited comparatively higher absorbance intensity than FD samples, indicating better retention of phenolics, betalains, flavonoids, and ascorbic acid. This may be due to rapid internal heating suggesting enhanced extractability and reduced drying time in MVD, which limited prolonged exposure to degradation conditions (31). Furthermore, 1 mm slices showed superior preservation compared to 2 mm slices in both drying methods due to faster moisture removal and lower internal diffusion resistance (32).

#### 3.3.2 Colour characteristics

The colour parameters of fresh beetroot were determined as lightness (L*) of 29.55, redness (a*) of 22.95 and yellowness (b*) of 5.4. The colour parameters (L, a and b) of MVD obtained beetroot powder were significantly influenced by the combined effect of microwave power, vacuum level and slice thickness (Figure 2). The lightness (L) values ranged from 18.93 to 34.87. Higher ‘L’ values were observed at 320 W with lower vacuum level (710 mm Hg), particularly in 2 mm slices, indicating greater puffing and porous structure formation due to rapid internal vapour generation during microwave heating. In contrast, lower microwave power combined with higher vacuum level (610 mm Hg) produced comparatively darker samples which can be attributes to a slower moisture removal promoting a structural shrinkage and concentration of pigments. The redness (a) values ranged from 17.00 to 27.20. The ‘a’ value, representing redness and betalain retention, was higher in samples dried at 320 W with lower to moderate vacuum levels, especially in 1 mm slices. Rapid volumetric heating at higher microwave power reduced drying time and minimized thermal degradation of betalain pigments, resulting in improved redness retention. However, under lower microwave power and higher vacuum conditions, prolonged drying exposure enhanced pigment degradation and oxidation, leading to lower ‘a’ value. The comparatively higher redness in thinner slices was attributed to faster moisture diffusion and reduced internal heat accumulation. Freeze drying exhibited the highest ‘a’ value due to sublimation based moisture removal at low temperature, which minimized thermal degradation of betalains. The yellowness (b) values varied from 4.20 to 12.37. The ‘b’ value increased under the combined conditions of lower microwave power, higher vacuum level and thicker slices, indicating increased yellowness and browning reactions. Prolonged drying under these conditions may have promoted non-enzymatic browning and degradation of red betalain pigments into yellowish compounds. In contrast, higher microwave power reduced browning due to shorter exposure time and rapid moisture removal.

**Figure 2:**
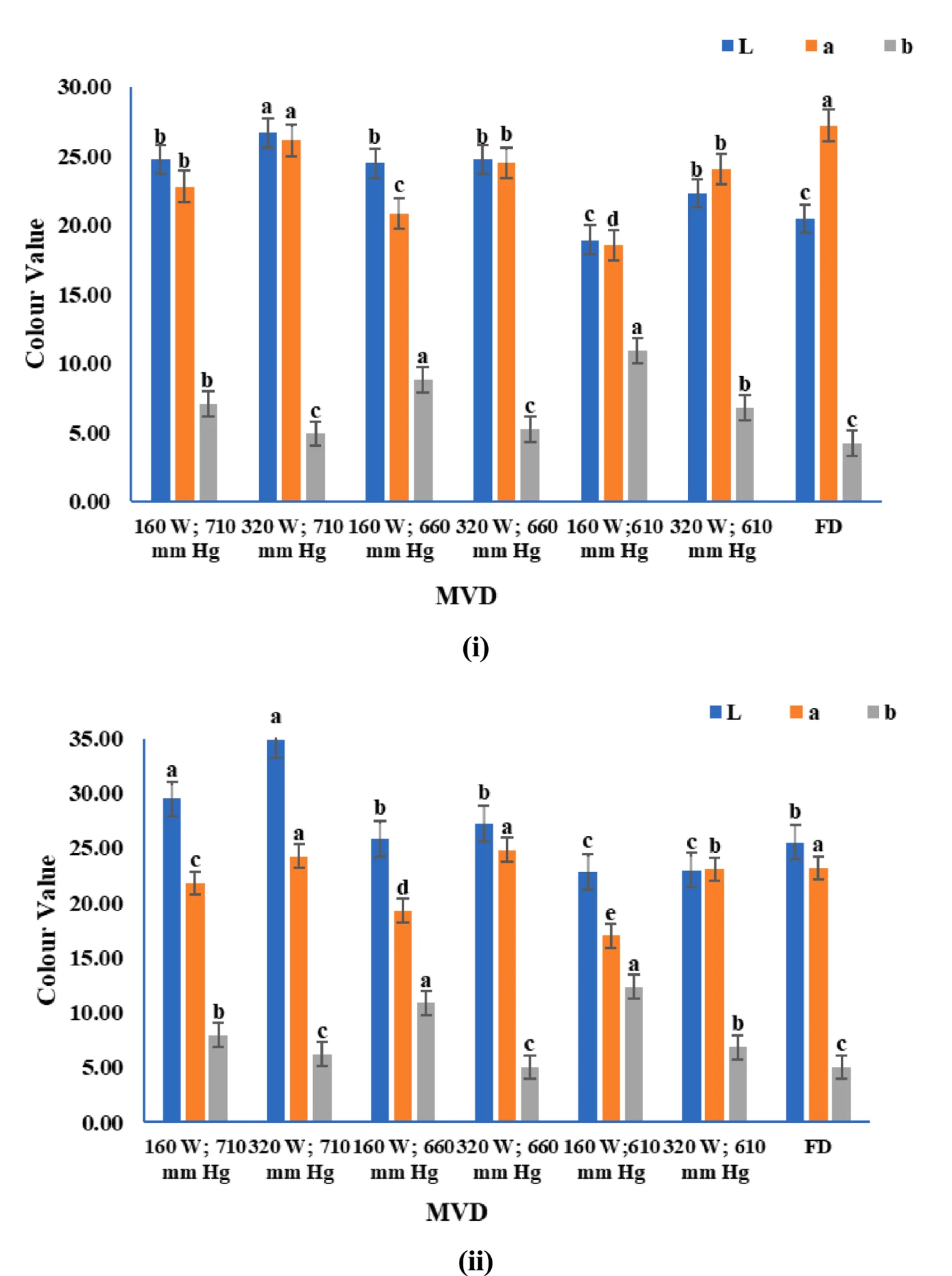
Colour values of beetroot powder as affected by microwave vacuum drying (MVD) and freeze drying (FD) for slice thickness **(i)** 1 mm, and **(ii)** 2 mm.

The total colour difference (∆E) and browning index (BI) of beetroot powder were significantly influenced by the combined effect of microwave power, vacuum level, slice thickness, and drying method. The ∆E values ranged from 4.50 to 11.22 for MVD samples and from 2.42 to 4.84 for (FD) samples (Table 2). Higher ∆E values indicated greater degradation of betalain pigments and deviation from the natural beetroot colour. The highest ∆E was observed in 1 mm slices dried at 160 W and 610 mm Hg, which may be attributed to prolonged drying duration and enhanced pigment degradation under low power conditions. In contrast, lower ∆E values at 320 W suggested improved colour retention due to rapid volumetric heating and shorter exposure time, which minimized thermal degradation of pigments. For the 2 mm slices, comparatively lower ∆E values at 320 W and 610 mm Hg indicated better colour stability under faster drying conditions. The BI values ranged from 65.62 to 147.49 for MVD samples and from 80.95 to 102.88 for FD samples. Higher BI values indicated increased non enzymatic browning and pigment deterioration during drying. The highest BI was observed at 160 W and 610 mm Hg for 1 mm slices, likely due to prolonged heat exposure promoting Maillard reactions and pigment oxidation. In contrast, lower BI values at higher microwave power, particularly at 320 W and 710 mm Hg for 2 mm slices, indicated reduced browning because rapid moisture removal limited thermal damage and browning reactions.

**Table 2:** Colour attributes of beetroot powder under varying drying condition and slice thickness.

Drying parameter		Slice thickness (mm)	Total colour difference (∆E)	Browning index (BI)
Power level (W)	Vacuum (mm Hg)			
Microwave vacuum drying				
160	710	1	6.99 ± 0.44ᵇ	93.01 ± 2.10ᵇ
320	710	1	5.90 ± 0.36ᶜ	82.11 ± 5.78ᶜ
160	660	1	7.63 ± 0.22ᵇ	100.47 ± 3.77ᵇ
320	660	1	5.62 ± 1.01ᶜ	86.27 ± 4.77ᶜ
160	610	1	11.22 ± 1.76ᵃ	147.49 ± 18.59ᵃ
320	610	1	6.87 ± 0.76ᵇ	103.99 ± 1.44ᵇ
160	710	2	6.60 ± 0.59ᵇ	80.49 ± 3.79ᶜ
320	710	2	10.60 ± 0.38ᵃ	65.62 ± 1.19ᵈ
160	660	2	4.70 ± 0.30ᶜ	104.32 ± 3.07ᵇ
320	660	2	7.11 ± 0.75ᵇ	79.93 ± 13.10ᶜ
160	610	2	4.86 ± 0.35ᶜ	126.08 ± 3.72ᵃ
320	610	2	4.50 ± 1.32ᶜ	99.20 ± 2.96ᵇ
Freeze drying (0.062 m bar, −40 °C)		1	4.84 ± 0.99ᶜ	102.88 ± 8.32ᵇ
		2	2.42 ± 1.21ᶜ	80.95 ± 13.74ᶜ

Different superscript small letters in each column denote significant difference at *p* ≤ 0.05.

Comparatively, FD samples exhibited lower ∆E values than most MVD treatments, confirming superior colour preservation under low-temperature drying conditions. However, optimized MVD treatments showed BI values comparable to FD samples, indicating effective control of browning under appropriate drying conditions. Overall, higher microwave power combined with moderate vacuum conditions improved colour retention and reduced browning by minimizing drying time and thermal degradation of betalain pigments (34). The L, a and b values, along with ΔE and BI, differed significantly among the drying treatments (*p* ≤ 0.05).

#### 3.3.3 Carr index and Hausner ratio

Powder properties influencing handling, packaging, and transportation, viz., bulk density, tapped density, true density, Carr Index (CI), Hausner ratio (HR), and bulk porosity, were significantly (*p* ≤ 0.05) affected by the combined effect of microwave power, vacuum level, slice thickness, and drying method (Table 3). In MVD samples, increasing microwave power from 160 to 320 W combined with higher vacuum level (610 mm Hg) and thinner slices (1 mm) resulted in lower bulk density (0.21 g/mL), lower true density (1.15 g/mL), and higher bulk porosity (78.42%), indicating enhanced puffing and formation of a highly porous structure due to rapid internal vapour generation during microwave heating. Similar trends were observed for 2 mm slices, where higher power and higher vacuum conditions produced lower density and higher porosity values. The increased porosity in high power MVD treatments also resulted in higher Carr Index (48–58%) and Hausner ratio (1.94–2.66), indicating poor flowability and greater cohesiveness of powders. This may be attributed to irregular particle structure, increased surface roughness and stronger inter particle friction caused by gross irregularity in the peripheral profile during rapid moisture evaporation and structural expansion (34). In contrast, samples processed at lower microwave power (160 W) and lower vacuum levels exhibited comparatively higher bulk density and lower porosity due to reduced puffing and compact particle formation.

**Table 3:** Powder properties of comminuted microwave vacuum and freeze dried beetroot slices of varying thickness.

Drying parameter		Slice thickness (mm)	Bulk density (g/mL)	Tapped density (g/mL)	True density (g/mL)	Hausner ratio (HR)	Carr index (%)	Bulk porosity (%)
Power level (W)	Vacuum (mm Hg)							
Microwave vacuum drying								
160	710	1	0.47 ± 0.01ᵃ	0.66 ± 0.02ᵇ	1.43 ± 0.11ᵃ	1.37 ± 0.02ᵇ	28.20 ± 1.20ᶜ	65.13 ± 5.80ᵇ
320	710	1	0.35 ± 0.00ᶜ	0.60 ± 0.01ᶜ	1.20 ± 0.01ᶜ	1.73 ± 0.04ᵃ	42.13 ± 1.18ᵇ	70.97 ± 0.23ᵃ
160	660	1	0.42 ± 0.00ᵇ	0.64 ± 0.00ᵇ	1.48 ± 0.02ᵃ	1.53 ± 0.01ᵇ	34.75 ± 0.32ᶜ	71.65 ± 0.24ᵃ
320	660	1	0.30 ± 0.02ᶜ	0.59 ± 0.00ᶜ	1.16 ± 0.01ᶜ	1.94 ± 0.10ᵃ	48.36 ± 2.72ᵇ	73.92 ± 1.41ᵃ
160	610	1	0.39 ± 0.00ᵇ	0.60 ± 0.01ᶜ	1.28 ± 0.00ᵇ	1.56 ± 0.01ᵇ	35.69 ± 0.41ᶜ	69.74 ± 0.04ᵇ
320	610	1	0.21 ± 0.02ᵈ	0.59 ± 0.02ᶜ	1.15 ± 0.01ᶜ	2.66 ± 0.59ᵃ	57.80 ± 14.60ᵃ	78.42 ± 7.29ᵃ
160	710	2	0.45 ± 0.08ᵃ	0.67 ± 0.02ᵇ	1.39 ± 0.21ᵃ	1.34 ± 0.01ᶜ	25.49 ± 0.54ᶜ	67.92 ± 1.72ᵇ
320	710	2	0.34 ± 0.01ᶜ	0.58 ± 0.00ᶜ	1.22 ± 0.01ᵇ	1.72 ± 0.03ᵇ	41.83 ± 0.93ᵇ	72.54 ± 0.29ᵃ
160	660	2	0.41 ± 0.01ᵇ	0.64 ± 0.00ᵇ	1.52 ± 0.01ᵃ	1.58 ± 0.01ᵇ	36.54 ± 0.44ᶜ	73.32 ± 0.25ᵃ
320	660	2	0.29 ± 0.00ᶜ	0.58 ± 0.00ᶜ	1.18 ± 0.01ᶜ	2.01 ± 0.01ᵃ	50.34 ± 0.26ᵇ	75.51 ± 0.26ᵃ
160	610	2	0.37 ± 0.00ᵇ	0.63 ± 0.01ᵇ	1.36 ± 0.01ᵃ	1.68 ± 0.01ᵇ	40.55 ± 0.25ᵇ	72.63 ± 0.25ᵃ
320	610	2	0.28 ± 0.00ᶜ	0.58 ± 0.00ᶜ	1.16 ± 0.01ᶜ	2.07 ± 0.03ᵃ	51.58 ± 0.64ᵇ	75.93 ± 0.31ᵃ
Freeze drying (0.062 mbar, −40 °C)		1	0.45 ± 0.00ᵃ	0.50 ± 0.00ᵈ	1.11 ± 0.00ᵈ	1.10 ± 0.00ᶜ	9.09 ± 0.00ᵈ	59.00 ± 0.00ᶜ
		2	0.63 ± 0.00ᵃ	0.71 ± 0.00ᵃ	1.66 ± 0.01ᵃ	1.14 ± 0.00ᶜ	12.50 ± 0.00ᵈ	63.00 ± 0.00ᶜ

Different superscript small letters in each column denote significant difference at *p* ≤ 0.05.

Compared to MVD powders, FD samples exhibited lower CI (9.09–12.50%) and HR (1.10–1.14), indicating superior flowability and lower cohesiveness. Freeze drying also produced comparatively denser and more uniformly packed particles with minimal structural collapse because moisture removal occurred through sublimation under low-temperature conditions. The uniform particle structure and reduced inter-particle friction contributed to improved flow behaviour of FD powders as has been reported while working with beetroot (19). Bulk density, tapped density, true density, Hausner ratio, Carr index, and bulk porosity differed significantly among the drying treatments (*p* ≤ 0.05).

#### 3.3.4 Reconstitution properties

There was a significant (*p* ≤ 0.05) combined effect of microwave power, vacuum level, slice thickness, and drying method on WAI and WSI of beetroot powder (Table 4). In MVD samples, WAI values ranged from 7.57 to 13.77 g/g, while WSI values ranged from 40.65 to 64.58%. Higher WAI values was observed in beetroot powder made from slices dried at lower microwave power (160 W) combined with thicker slices (2 mm), particularly at 660 and 710 mm Hg, indicating better water absorption due to reduced structural incoherence and preservation of cellular integrity during slower drying. In contrast, higher microwave power (320 W) combined with higher vacuum level (610 mm Hg) resulted in lower WAI values, suggesting greater structural disruption and collapse caused by rapid moisture evaporation and intense volumetric heating.

**Table 4:** Reconstitution properties of beetroot powder under varying drying condition and slice thickness.

Drying parameter		Slice thickness (mm)	WAI (g/g of dry matter)	WSI (%)
Power level (W)	Vacuum (mm Hg)			
Microwave vacuum drying				
160	710	1	11.99 ± 1.65ᵃ	48.47 ± 0.36ᶜ
320	710	1	9.07 ± 0.06ᶜ	62.42 ± 0.57ᵇ
160	660	1	10.83 ± 0.06ᵇ	50.46 ± 0.47ᶜ
320	660	1	8.68 ± 0.04ᶜ	64.09 ± 1.37ᵇ
160	610	1	9.95 ± 0.04ᵇ	52.46 ± 0.40ᶜ
320	610	1	7.57 ± 0.12ᶜ	64.58 ± 5.81ᵇ
160	710	2	13.59 ± 1.89ᵃ	40.65 ± 0.14ᶜ
320	710	2	10.07 ± 0.05ᵇ	54.16 ± 0.81ᵇ
160	660	2	13.77 ± 0.30ᵃ	44.49 ± 0.16ᶜ
320	660	2	9.61 ± 0.08ᵇ	53.27 ± 0.38ᵇ
160	610	2	11.35 ± 0.20ᵇ	46.57 ± 0.68ᶜ
320	610	2	8.24 ± 0.14ᶜ	59.12 ± 0.56ᵇ
Freeze drying (0.062 mbar, −40 °C)		1	10.34 ± 1.11ᵇ	55.53 ± 1.01ᶜ
		2	13.49 ± 0.35ᵃ	44.67 ± 2.52ᶜ

Different superscript small letters in each column denote significant difference at *p* ≤ 0.05.

The WSI values increased for beetroot powder from slices exposed to a combined condition of higher microwave power, higher vacuum level, and thinner slices. The maximum WSI was observed in powder made from 1 mm thick slices dried at 320 W and 610 mm Hg, indicating enhanced solubilization due to porous structure formation and breakdown of macromolecular components during rapid drying. Lower WSI values was observed at 160 W in powder made from thicker slices indicating a comparatively lower solubilization because slower drying promoted retention of intact cellular structure and stable molecular interactions (35).

Freeze-dried powder samples exhibited comparatively higher WAI and moderate WSI values than most high-power MVD treatments. The higher WAI in FD powders may be attributed to the porous and less collapsed structure formed during sublimation drying, which improved water absorption capacity. Meanwhile, moderate WSI values indicated better structural integrity and lower degradation of soluble components compared to MVD powders. Similar powder behaviour was reported in case of blueberry by (36). WAI and WSI differed significantly among the drying treatments (*p* ≤ 0.05).

### 3.4 Biochemical properties

#### 3.4.1 Proximate analysis

Powder from slices dried at lower microwave power (160 W) combined with higher vacuum level (610 mm Hg) and thicker slices (2 mm) showed maximum fibre (2.37%), protein (11.86%), fat (2.30%), and ash (8.26%) contents (Table 5).

**Table 5:** Compositional details of beetroot powder under varying drying condition and slice thickness.

Drying parameter		Slice thickness (mm)	Fibre (%)	Fat (%)	Protein (%)	Ash (%)	Carbohydrate (%)	Energy (kcal)
Power level (W)	Vacuum (mm Hg)							
Microwave vacuum drying								
160	710	1	1.49 ± 0.02ᶜ	1.10 ± 0.12ᵇ	7.63 ± 0.09ᶜ	4.47 ± 0.33ᶜ	82.88 ± 0.36ᵃ	371.98 ± 2.13ᵃ
320	710	1	1.41 ± 0.01ᶜ	0.87 ± 0.02ᶜ	7.05 ± 0.05ᵈ	5.13 ± 0.03ᵇ	83.77 ± 0.05ᵃ	371.16 ± 0.17ᵃ
160	660	1	1.69 ± 0.08ᵇ	1.82 ± 0.02ᵃ	8.89 ± 0.13ᵇ	6.92 ± 0.04ᵇ	79.03 ± 0.14ᵇ	368.04 ± 0.27ᵇ
320	660	1	1.65 ± 0.25ᵇ	1.30 ± 0.01ᵇ	7.85 ± 0.03ᶜ	6.70 ± 0.10ᵇ	81.08 ± 0.12ᵃ	367.45 ± 0.27ᵇ
160	610	1	2.08 ± 0.09ᵃ	2.01 ± 0.01ᵃ	10.63 ± 0.20ᵃ	8.09 ± 0.10ᵃ	74.47 ± 0.49ᶜ	358.43 ± 1.27ᶜ
320	610	1	1.82 ± 0.35ᵇ	1.61 ± 0.37ᵇ	8.73 ± 0.53ᵇ	8.08 ± 0.02ᵃ	77.09 ± 0.15ᵇ	357.74 ± 1.90ᶜ
160	710	2	1.61 ± 0.03ᶜ	1.62 ± 0.22ᵇ	7.80 ± 0.58ᶜ	6.19 ± 0.04ᵇ	80.29 ± 0.07ᵃ	366.97 ± 4.49ᵃ
320	710	2	1.50 ± 0.02ᶜ	0.97 ± 0.03ᶜ	7.34 ± 0.02ᶜ	5.82 ± 0.41ᵇ	82.78 ± 0.38ᵃ	369.20 ± 1.71ᵃ
160	660	2	1.86 ± 0.06ᵇ	1.79 ± 0.02ᵃ	10.21 ± 0.01ᵃ	7.19 ± 0.06ᵃ	76.63 ± 2.36ᵇ	363.43 ± 9.42ᵇ
320	660	2	1.64 ± 0.03ᵇ	1.44 ± 0.03ᵇ	8.47 ± 0.02ᵇ	7.13 ± 0.97ᵃ	78.47 ± 0.31ᵇ	360.69 ± 1.38ᵇ
160	610	2	2.37 ± 0.05ᵃ	2.30 ± 0.02ᵃ	11.86 ± 0.03ᵃ	8.26 ± 0.12ᵃ	72.99 ± 0.20ᶜ	360.09 ± 0.55ᵇ
320	610	2	2.26 ± 0.05ᵃ	1.84 ± 0.07ᵇ	9.48 ± 0.27ᵇ	8.47 ± 0.14ᵃ	75.99 ± 0.27ᵇ	358.41 ± 0.38ᵇ
Freeze drying (0.062 mbar, −40 °C)		1	1.85 ± 0.02ᵇ	1.44 ± 0.27ᵇ	10.16 ± 0.43ᵃ	7.69 ± 0.27ᵃ	76.49 ± 0.44ᵇ	359.54 ± 2.34ᶜ
		2	2.11 ± 0.08ᵃ	1.38 ± 0.12ᵇ	9.84 ± 0.63ᵇ	8.26 ± 0.12ᵃ	75.28 ± 0.42ᵇ	352.87 ± 0.44ᶜ

Different superscript small letters in each column denote significant difference at *p* ≤ 0.05.

Similar trends were observed for 1 mm slices at the same drying conditions. The higher retention of these components may be attributed to concentration effects caused by greater moisture removal and reduced dilution of nutrients during drying (37). In contrast, samples processed at higher microwave power (320 W) combined with lower vacuum levels (710 mm Hg) exhibited higher carbohydrate content (82–84%) and energy values (369–372 kcal). Rapid drying under these conditions likely minimized degradation of soluble carbohydrates and reduced structural concentration of other constituents. However, higher microwave power combined with higher vacuum conditions during slice drying slightly reduced protein and fat contents, possibly due to thermal degradation and oxidation during intense volumetric heating Slice thickness also influenced nutrient composition of beetroot powder, where 2 mm slices contained slightly higher fibre, protein, and ash than 1 mm slices powder, this may be due to slower moisture removal and greater concentration of solids during drying. Fibre, fat, protein, ash, carbohydrate, and energy content differed significantly among the drying treatments (*p* ≤ 0.05).

Powder from FD samples exhibited comparatively higher protein and ash retention with moderate carbohydrate and energy values, indicating better preservation of nutritional quality under low-temperature sublimation drying conditions.

#### 3.4.2 Total flavonoid content and antioxidant activity

Processing parameters of the beetroot slices has significant (*p* ≤ 0.05) effect on the antioxidant activity (%RSA) and total flavonoid content of the beetroot powder (Figures 3, 4).

**Figure 3:**
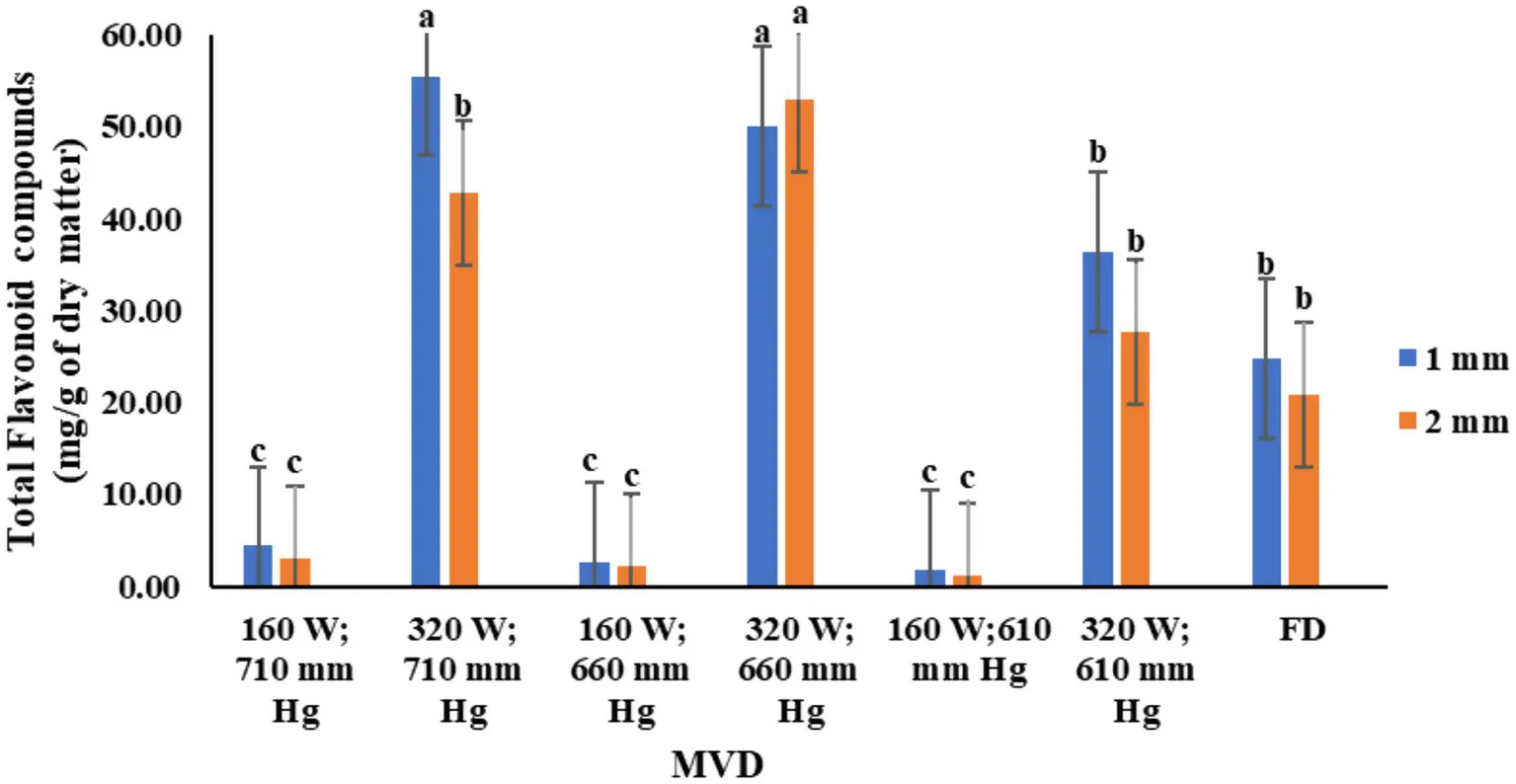
Total flavonoid content of beetroot powder from different slice thickness under different drying conditions.

**Figure 4:**
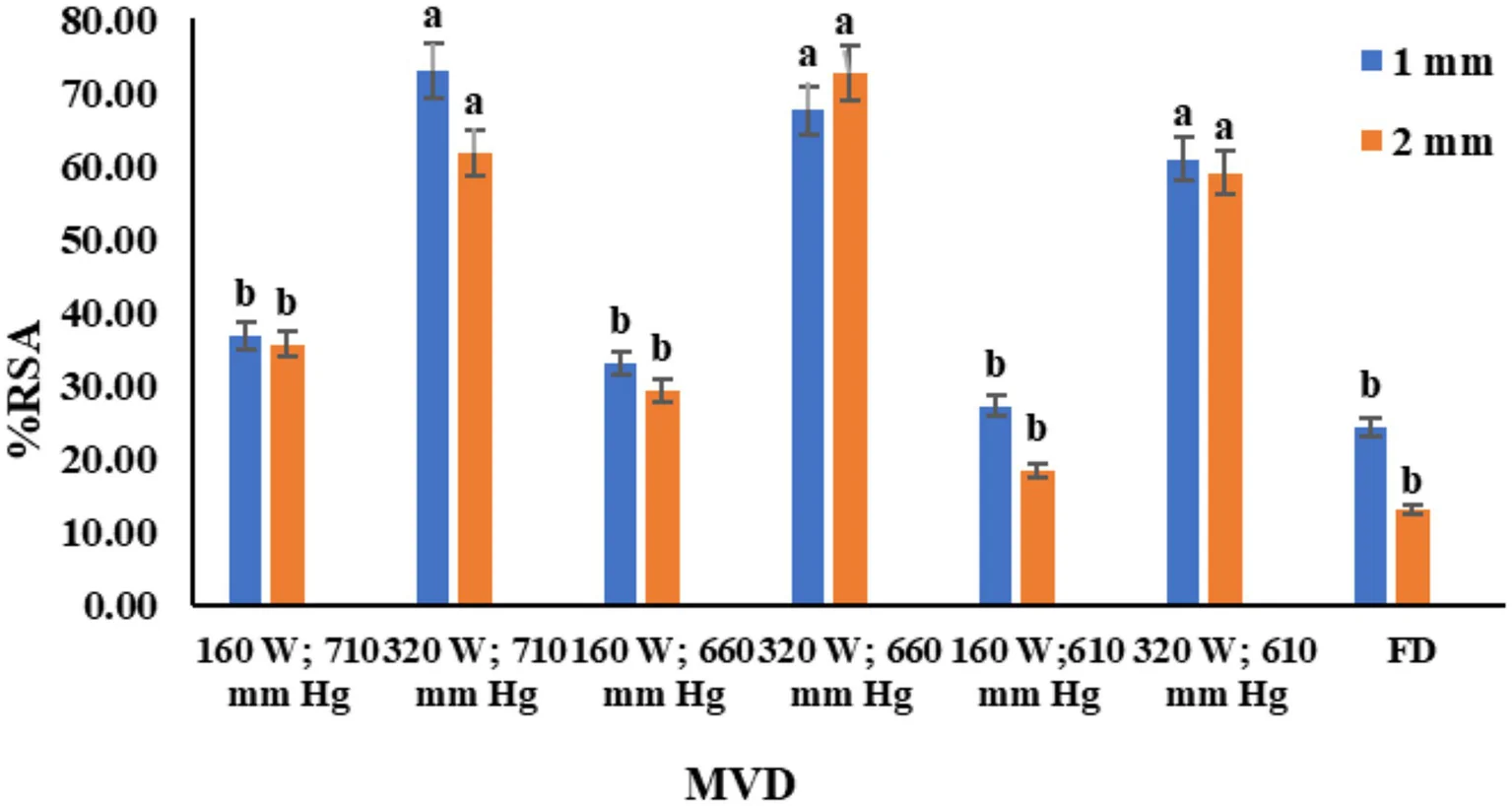
Free radical scavenging activity (RSA, %) of beetroot powder under drying conditions.

In MVD samples, higher microwave power (320 W) combined with lower to moderate vacuum levels (710 to 660 mm Hg) resulted in maximum %RSA and TFC irrespective of the slice thickness of beetroot. The highest TFC was observed for the sample made at 320 W and 710 mm Hg with 1 mm slices, while maximum antioxidant activity was recorded at 320 W and 660 mm Hg with 2 mm slices. The improved retention of flavonoids and antioxidant activity under these conditions may be attributed to rapid volumetric heating and shorter drying duration, which minimized thermal degradation and enhanced release of bound phenolic compounds through cell structure disruption (26). In contrast, lower microwave power (160 W) combined with higher vacuum level (610 mm Hg) resulted in the lowest %RSA and TFC values, indicating greater degradation of antioxidant compounds due to prolonged drying and oxidative deterioration of the slices. Powder made from thinner slices (1 mm) generally exhibited slightly higher antioxidant activity and flavonoid retention than 2 mm slices because faster moisture diffusion reduced internal heat exposure and degradation of thermosensitive compounds.

Powder of freeze drying origin had a comparatively lower %RSA and moderate TFC values than optimized MVD treatments. Although freeze drying effectively preserved flavonoids due to low temperature drying, the antioxidant activity remained lower, possibly because of sublimation during freeze drying preserved the compounds without significantly enhancing their extractability (28). TFC and %RSA content differed significantly among the drying treatments (*p* ≤ 0.05).

#### 3.4.3 Betalain

Powder from slices dried at higher microwave power (320 W) combined with lower to moderate vacuum levels (710 to 660 mm Hg) resulted in comparatively higher retention of total betalain, particularly in 1 mm slices. Rapid volumetric heating under these conditions shortened drying time and reduced pigment exposure to thermal degradation, while porous structure formation enhanced moisture removal and pigment stability. In contrast, lower microwave power (160 W) combined with higher vacuum level (610 mm Hg) showed the lowest betalain retention due to prolonged drying and greater degradation of pigment molecules. Slice thickness also affected pigment stability, where 1 mm slices generally retained higher betalain content than 2 mm slices because faster moisture diffusion reduced internal heat accumulation and oxidative degradation (Figures 5i,ii). Betanin showed greater degradation than betaxanthin under severe drying conditions, indicating its comparatively lower thermal stability (28, 34). Overall, the results demonstrated that betalain retention was strongly governed by the interaction between drying rate, vacuum conditions, and slice thickness. Freeze drying provided maximum pigment preservation, whereas optimized MVD conditions involving higher microwave power, lower to moderate vacuum level, and thinner slices improved betalain retention by balancing rapid drying with reduced thermal damage.

**Figure 5:**
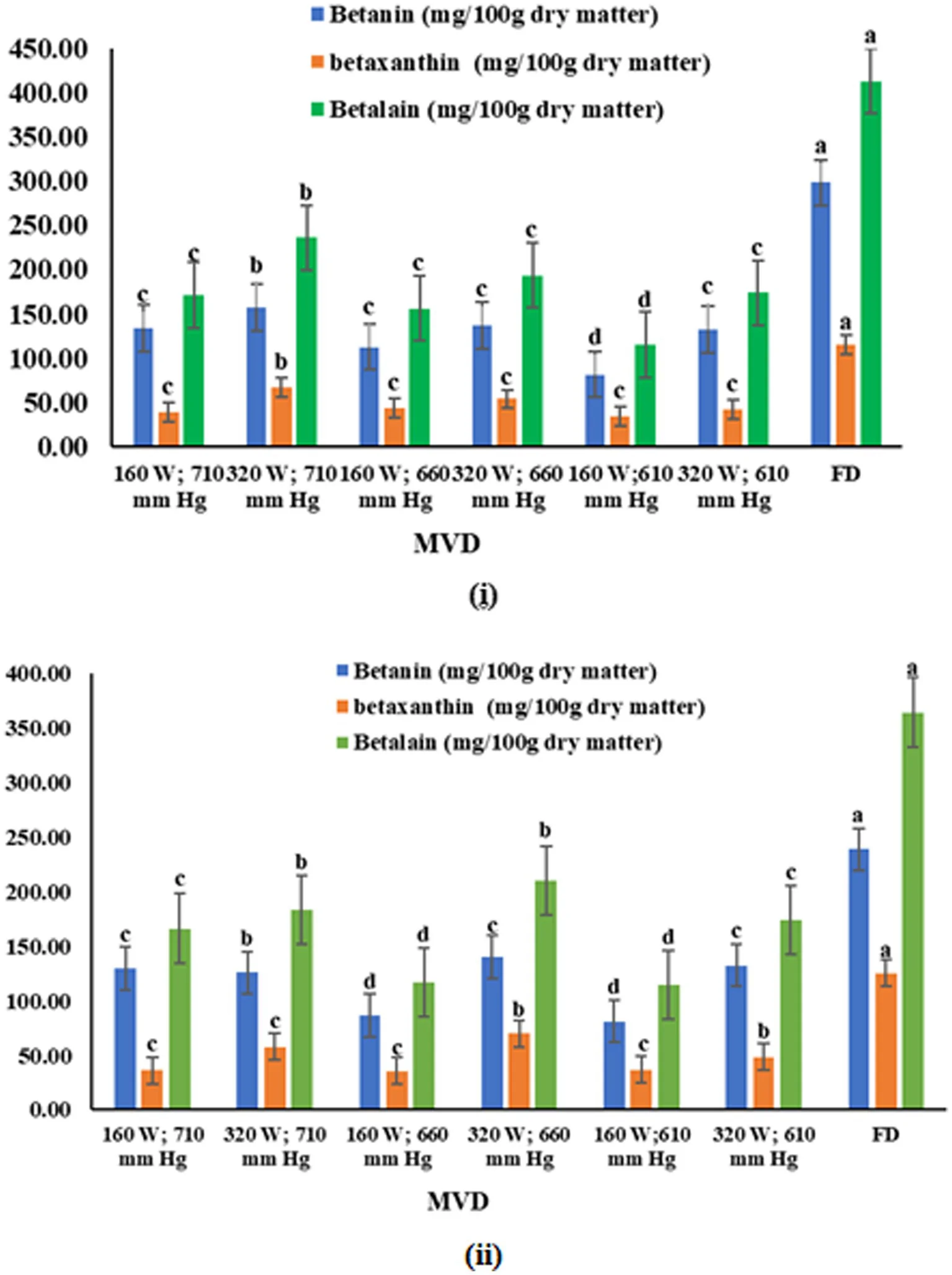
Betalains content of beetroot powder under different drying conditions made from slice thickness **(i)** 1 mm, and **(ii)** 2 mm.

Freeze dried samples showed the highest retention of total betalain for both slice thicknesses, with total betalain values reaching about 364 to 413 mg/100 g dry matter (Figures 5i and ii). The superior pigment retention in FD samples may be attributed to low-temperature sublimation drying, which minimized thermal and oxidative degradation of betalain molecules and preserved cellular structure. Betanin, betaxanthin, and total betalain contents differed significantly among the drying treatments (*p* ≤ 0.05), indicating that microwave power, vacuum level, and slice thickness significantly influenced the retention of betalain pigments in beetroot powder.

## 4 Conclusion

This study demonstrated that microwave vacuum drying (MVD) significantly influences physicochemical properties and bioactive retention of beetroot powder. MVD reduced drying time compared to freeze drying (FD), highlighting its efficiency as a rapid dehydration technique. Colour attributes, FTIR analysis and pigment retention evaluation confirmed that drying conditions strongly affect structural integrity and phytochemical stability. Optimized MVD conditions, particularly higher microwave power (320 W) combined with modest vacuum levels (≤660 mm Hg) and thinner slices (1 mm), resulted in improved redness (a), betalain pigments retention and preservation of key functional groups, due to rapid moisture removal and reduced exposure to degrading environments. While, lower power and higher vacuum levels led to prolonged drying, structural degradation, increased browning, and significant loss of bioactive compounds. MVD produced powder with low bulk density, high porosity and favourable reconstitution behaviour, characteristics that support dispersibility and solubility but resulted in poor flow property, as reflected by higher Carr index and Hausner ratio. On the other hand, FD produced powders exhibited superior flow properties due to smoother and more uniform particle structure. Functional properties, i.e., WAI and WSI indicated that higher power enhanced solubility but reduced water absorption capacity which, reflects structural breakdown of the matrix.

Bioactive analysis describes that betalain pigments and total flavonoids were best preserved under FD, due to low-temperature sublimation and minimal thermal stress on thermosensitive compounds. However, optimized MVD conditions (320 W, ≤660 mm Hg, 1 mm thickness) achieved comparatively high retention by balancing drying rate and thermal exposure. Structural stability of betalains was closely linked to drying conditions, as thermal degradation mechanisms such as chromophore breakdown and oxidation were minimized under rapid drying. Also, the interaction between microwave power and vacuum level markedly influenced nutrient distribution in the beetroot powders, where lower microwave power with higher vacuum level promoted concentration of fibre, protein, fat, and ash, whereas higher microwave power with lower vacuum level favoured carbohydrate retention and higher energy values.

Collectively, these findings indicate that FD remains superior for maximum quality retention but MVD offers a promising alternative due to its significantly reduced drying time and acceptable preservation of colour, structure and bioactive compounds. Optimization of process parameters in MVD such as microwave power, vacuum level and slice thickness is critical to achieving high quality dried beetroot powder with enhanced functional and nutritional properties suitable for use as a natural colourant and value-added ingredient in sustainable food systems. Future studies should investigate the effects of storage and packaging conditions on pigment stability in food matrices and other intended applications, thereby supporting the selection of appropriate drying techniques based on specific end use requirements.

## Data Availability

The original contributions presented in the study are included in the article/supplementary material, further inquiries can be directed to the corresponding author/s.
